# A Rare Case of an Azygos Lobe in the Right Lung of a 40-year-old Male

**DOI:** 10.7759/cureus.2780

**Published:** 2018-06-11

**Authors:** Georgi Kotov, Iva N Dimitrova, Alexandar Iliev, Violeta Groudeva

**Affiliations:** 1 Department of Anatomy, Histology and Embryology, Medical University of Sofia, Sofia, BGR; 2 Department of Cardiology, University Hospital St. Ekaterina, Medical University of Sofia, Sofia, BGR; 3 Department of Diagnostic Imaging, Medical University of Sofia, Sofia, BGR

**Keywords:** azygos lobe, lung, radiology, variation

## Abstract

The azygos lobe is a rare anatomical variant, most often encountered in the right lung. Its etiology is related to a defect of the migration of the azygos vein during the embryonic development. Here, we describe a rare case of an azygos lobe of the right lung, diagnosed incidentally on a computed tomography (CT) scan in a 40-year-old male patient who presented with chest pain, shortness of breath, and fever. The initial differential diagnosis included acute myocardial infarction, aortic dissection, and myocarditis. Chest CT with contrast matter demonstrated a peculiar finding in the right lung which was recognized as an azygos vein passing through the upper lobe and separating an azygos lobe with its mesoazygos. The azygos lobe may be rarely associated with neoplastic processes or spontaneous pneumothorax and its differential diagnosis on imaging studies includes various conditions. Knowledge of this variation is important to avoid misdiagnosis or complications during thoracic surgery.

## Introduction

The azygos lobe is a rare anatomical variant, most often encountered in the right lung [1-2]. Its prevalence in the clinical setting is 0.4%, and 1% in anatomical dissections [1]. Normally, the thoracic portion of the azygos vein is derived from the right posterior cardinal vein and arches over the apex of the right lung, draining towards the superior vena cava [1]. In some cases, however, the vein penetrates through the upper lobe of the right lung and drags the parietal and visceral pleura with it, thus creating an accessory fissure, known as the ‘azygos fissure’, which can have either a vertical or an oblique course [1,3]. The azygos vein, in this case, is found passing along the bottom of the fissure and is suspended from the thoracic wall through a fold of the parietal pleura, called the mesoazygos [1-2]. This leads to the detachment of the superior-medial portion of the upper lobe of the lung, located above the hilum and medial to the accessory fissure, which is referred to as ‘azygos lobe’ [1,4]. In truth, the azygos lobe is not an independent segment of the right lung; hence the term 'lobe' is not entirely correct [2].

Clinically, the azygos lobe is a variation that can simulate various diseases and is most often found incidentally during chest radiography or computed tomography (CT) [5-6]. Its radiographic appearance is that of a dense, comma-shaped shadow which begins at the apex of the right lung and curves downwards and inwards towards the mediastinum, ending a little below the level of the first costal cartilage [1]. Here, we describe a rare case of an azygos lobe of the right lung, diagnosed incidentally on a CT scan in a 40-year-old male patient.

## Case presentation

**Figure 1:**
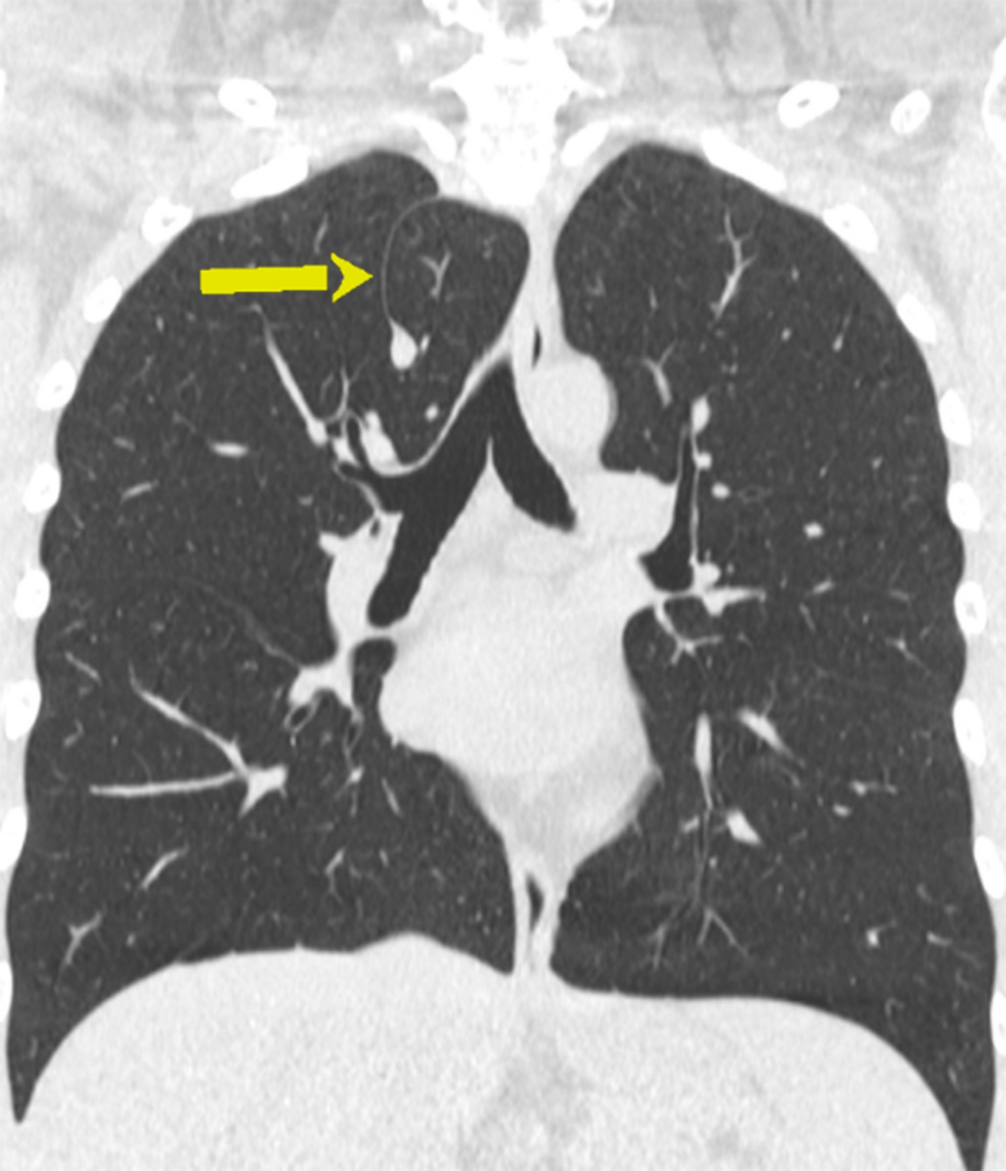
Computed tomography (CT) of the chest - coronal image; azygos lobe (arrow)

**Figure 2:**
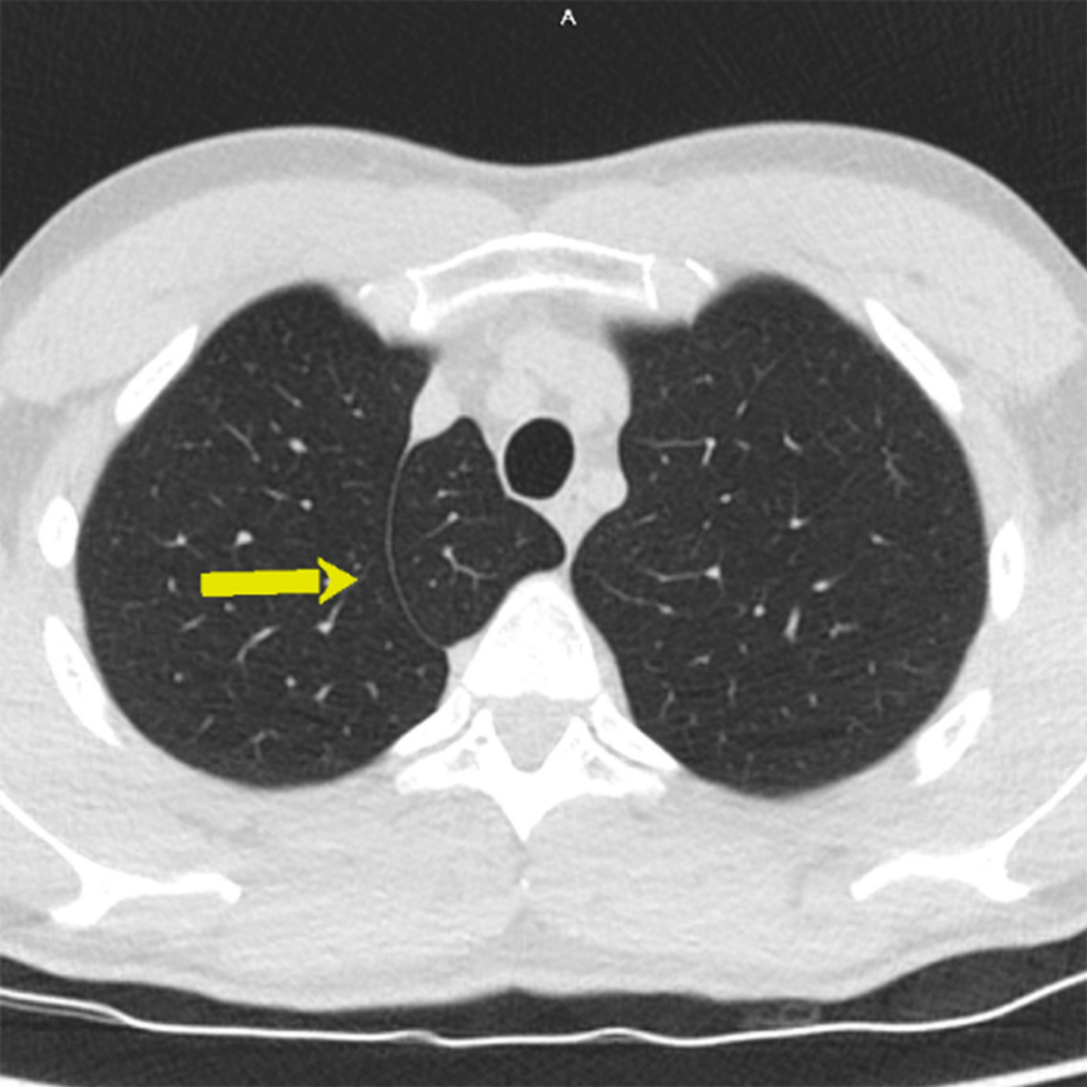
Computed tomography (CT) of the chest - axial image; azygos lobe (arrow)

A 40-year-old male of Caucasian race presented with chest pain propagating to the left arm and the back and shortness of breath. These symptoms started on the day prior to the admission and were preceded by high-grade fever. The vitals were normal. Cardiac examination was normal. The electrocardiogram showed diffuse repolarisation changes. Transthoracic echocardiography revealed impaired systolic function of the left ventricle with ejection fraction 48%. Cardiac enzymes - creatine phosphokinase (CPK), creatine phosphokinase myocardial band fraction (CPK-MB), and troponin T were all markedly elevated. The blood lipid tests were within reference ranges. The initial differential diagnosis included acute myocardial infarction, aortic dissection, and myocarditis. CT of the chest with contrast matter showed no signs of aortic dissection. A peculiar finding was noted in the right lung and was recognized as an azygos vein passing through the upper lobe and separating an azygos lobe with its mesoazygos (Figures 1-2).

The patient was evaluated through coronary angiography which did not show any evidence of coronary artery disease or myocardial infarction. Finally, microbiological tests were conducted and revealed Epstein-Barr virus (EBV) infection, which was discussed as the cause of acute myocarditis. The patient was treated accordingly and returned to normal activity within a few days.

## Discussion

The present case report describes a rare anatomical variation of the right lung, found incidentally on a CT scan in a 40-year-old male patient with acute myocarditis. This anomalous structure did not present with any clinical symptoms. On imaging studies, the azygos lobe may mimic an enlarged thymus, a substernal goiter, a localised pneumothorax, bulla, lung abscess, or neoplasm [2,6-7]. Diagnosing the azygos lobe may be further complicated by physiological changes in the size of the azygos vein or morphological variants of the fissure [8]. Three types of azygos fissure have been described [3]. Type A is a more or less horizontal fissure which cuts the lateral portion of the lung between the apex and a point located 2 cm above. Type B is a vertical fissure dividing the apex into two lateral halves. Type C is a vertical fissure which starts from the mediastinal aspect of the lung and cuts off a small portion of the upper lobe which is fixed above the root of the lung. Our case was consistent with Type C. Ndiaye et al. also reported that a very deep fissure may compress the underlying bronchus draining the azygos lobe and thus lead to atelectasis or bronchiectasis [3]. Such findings were not observed in the present case.

Despite mostly considered as a benign anomaly in the lung anatomy, the clinical significance of the azygos lobe in the case of pathological conditions of the lung has been broadly discussed. One study reported that the azygos lobe may be isolated from pathological processes developing in the rest of the lung tissue due to the presence of the mesoazygos [8]. For instance, dissemination of pulmonary tuberculosis, which most often involves the apex of the lung, into the azygos lobe is an uncommon finding [8]. Conversely, pathological processes arising in the azygos lobe may be confined to it. Denega et al. reported that carcinoma of the azygos lobe is not associated with regional lymph node involvement [8]. This was confirmed by Darlong et al. who reported a squamous cell carcinoma arising in the azygos lobe with negative mediastinal lymph nodes [6]. Fukuhara et al. described a case of a solitary nodule in an azygos lobe of the right lung, 2 cm in diameter, verified as an adenocarcinoma, again without any metastatic involvement of the regional lymph nodes and treated successfully with robot-assisted thoracic surgery [4]. Another study reported that the azygos lobe is very rarely associated with spontaneous pneumothorax and may even play a somewhat protective role against the formation of bullae and blebs in the apex, which is often observed in young individuals presenting with spontaneous pneumothorax [9]. The anomalous course of the azygos vein may complicate surgical interventions or the evolution of infiltrative processes in the upper lobe. Sometimes, aneurysms of the vein may develop and lead to symptoms of pressure or tightness in the chest [8].

## Conclusions

The azygos lobe is a rare anatomical variant resulting from a failure in the migration of the azygos vein during the embryonic development. Although rarely associated with pathological conditions, it may be an accidental finding on imaging studies, such as the case presented here. Knowledge of this variation is important in order to prevent misdiagnosis and/or unnecessary interventions.
